# Possibilities of a Hybrid Method for a Time-Scale-Frequency Analysis in the Aspect of Identifying Surface Topography Irregularities

**DOI:** 10.3390/ma16031228

**Published:** 2023-01-31

**Authors:** Damian Gogolewski, Paweł Zmarzły, Tomasz Kozior, Thomas G. Mathia

**Affiliations:** 1 Department of Mechanical Engineering and Metrology, Kielce University of Technology, al. Tysiąclecia Państwa Polskiego 7, 25-314 Kielce, Poland; 2 Laboratoire de Tribologie et Dynamique des Systemes (LTDS), Ecole Centrale de Lyon, Centre National de la Recherche Scientifique, 69134 Lyon, France

**Keywords:** wavelet transformation, Fourier analysis, surface texture, rolling bearings, roundness, surface quality

## Abstract

The article presents research results related to assessing the possibilities of applying modern filtration methods to diagnosing measurement signals. The Fourier transformation does not always provide full information about the signal. It is, therefore, appropriate to complement the methodology with a modern multiscale method: the wavelet transformation. A hybrid combination of two algorithms results in revealing additional signal components, which are invisible in the spectrum in the case of using only the harmonic analysis. The tests performed using both simulated signals and the measured roundness profiles of rollers in rolling bearings proved the advantages of using a complex approach. A combination of the Fourier and wavelet transformations resulted in the possibility to identify the components of the signal, which directly translates into better diagnostics. The tests fill a research gap in terms of complex diagnostics and assessment of profiles, which is very important from the standpoint of the precision industry.

## 1. Introduction

Metrology 4.0 is undoubtedly an important component of the fourth industrial revolution which is entering a dynamic phase. Measurements and a proper analysis of the acquired data constitute an integral part of the process and the functioning of systems. As part of Metrology 4.0, a number of measurement devices have been developed and improved, which can execute requested processes in an automatic manner in real-time using artificial intelligence and make decisions in an autonomous way, based on the acquired data. In recent years, new solutions were also introduced in the aspect of surface topography, allowing for a more detailed and comprehensive assessment and prediction [1,2]. Modern multiscale methods constitute an important contribution to the development of modern surface metrology, and they contribute to a considerable increase in the possibilities of its assessment [3,4]. When surfaces are observed on numerous scales, there is a possibility to observe and highlight their characteristic features. The surface texture of the produced elements is described as a set of periodic and aperiodic irregularities. This distribution depends on the procedures to which the surface was subjected, while taking into account the parameters of specific machining processes. There are a number of algorithms used to assess, filtrate, or detect certain important information included in such surfaces; however, the application of traditional algorithms often does not allow for the identification of all the components of a signal. It is, therefore, desirable to eliminate the shortcomings of the individual transforms and to analyze the measurement data in a more efficient way. The hybrid application of several known methods in a proper order allows for a better, comprehensive evaluation of the signals.

The nature of irregularities of rotating elements is evaluated using a roundness profile transformation based on the Fourier transformation. In accordance with this approach, the individual profiles are described as a composition of periodic functions with a defined magnitude of the amplitude and period, which provide information on possible errors in the manufacturing process. Based on the magnitude of the individual harmonic components, it is possible to draw a conclusion about the prevalent deviations of the shape profile and about their potential impact on the subsequent work of an element. Nonetheless, in the case of local defects existing on the surface, a harmonic analysis does not provide a complete image. The values of the individual components of the signal are considerably disrupted by scratches or cracks. As a supplement to the Fourier transformation, one can suggest the concept of using an additional method developed at the turn of the 21st century: the wavelet transformation. 

The wavelet transformation is one of the multiscale methods which allow for decomposing a signal and presenting it in individual ranges. Currently, it is also increasingly used to analyze 2D and 3D surface profiles. These profiles are a composition of periodic and aperiodic irregularities; therefore, they should be assessed as non-stationary signals and analyzed with the use of proper methods. Such methods include wavelet transformation, allowing for simultaneous localization in the time and frequency domain, which is its unquestionable advantage. However, this transformation also has disadvantages, such as, among other things, shift sensitivity, poor directionality, and the lack of phase information [5]. The discrete wavelet transform used in the study is based on the use of two types of filters, i.e., high-pass and low-pass. The particular combination of them allows for the evaluation of surface topography in a specific frequency band, the size of the scale. In addition, a wide spectrum of mother wavelets with different properties allows for their appropriate use, which translates into better diagnostics and detection of characteristic features of the signal which are invisible in the results of the analysis carried out by other methods. When analyzing the literature, it can be concluded that in surface metrology, a number of uses have already been found for the wavelet transformation, e.g., for correcting temperature errors [6], diagnosing, characterizing, and identifying characteristic features [7,8,9], decomposition the profile into components (roughness, waviness, form) [10,11,12], and estimating surface roughness parameters using image processing [13]. 

**Figure 1:**
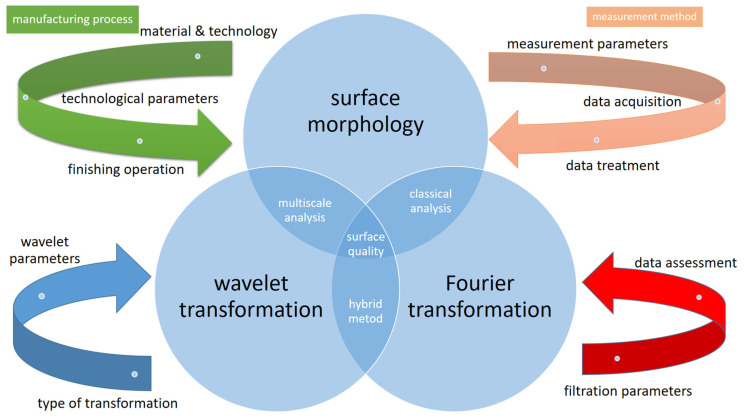
Relationship between surface morphology and methods for analyzing measurement data.

Therefore, it was desirable to compare the possibilities of using the developed wavelet approach and Fourier transformation in the aspect of analyzing various types of signals. Research pointing to the drawbacks and advantages of specific methods using the example of the surface profiles of rotating elements are described in [14]. In [15], the authors compare both methods in the aspect of assessing muscle fatigue, while in [16], the Fourier transform, the windowed Fourier transform, and the wavelet transform methods were applied to fringe pattern processing. Research intended to identify the error components influencing the measurement accuracy of the system using the Fourier transformation and the wavelet transformation is presented in the paper [17]. Based on the current state of the art, it should be concluded that both methods can complement each other; therefore, a proper combination of both methods can be used successfully for proper diagnostics of signals. A proper combination of both algorithms has been used to identify defects of bearings in [18,19,20]. Nonetheless, the use of a hybrid method based on the time-scale-frequency analysis to diagnose measurement signals provides potentially vast possibilities, and it requires a broader analysis. The novelty of the work is the application of a comprehensive approach to the evaluation of surfaces using the modern, multiscale, hybrid method including the analysis of its parameters. The tests fill a research gap in terms of complex diagnostics and assessment of profiles. Despite the many studies relating to the evaluation of wavelet transformation parameters and their impact on results, further comprehensive analysis of their applicability is required [21,22,23,24]. The relationship between the surface texture, which is influenced by the parameters of both the process and the measurement, and the methods of analysis, is very important in the context of a comprehensive analysis of surface quality, as shown in Figure 1. It should be pointed out that there is no research aimed at assessing the possibilities of using the wavelet transform to analyze the roundness profiles, in particular in the elements of rolling bearings. It should be added that this type of shape deviation has a considerable impact on the operating parameters of rolling bearings, and new methods of their assessment should be sought [25]. 

Figure 1 presents a schematic interpretation of the production and measurement process, taking into account the technological [26,27,28,29,30,31] and measuring process [32,33,34,35,36,37,38,39], with particular emphasis on the wavelet [40,41,42,43] and Fourier transformation [44,45,46,47,48,49].

## 2. Metrological Approach

**Figure 2:**
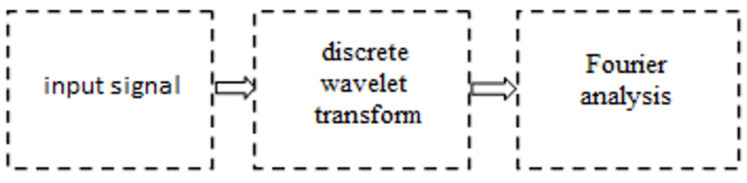
Diagram of the hybrid method of the time-scale-frequency analysis.

This paper presents simulation tests intended to assess the possibilities of identifying signal disruptions using a time-scale-frequency analysis (Figure 2). The hybrid approach methodology is based on the performance of a spectral analysis of signals generated at the subsequent stages of the wavelet decomposition. Assessment of the signal, taking into account the scale of irregularities, will allow for the identification of disruptions that are invisible in the input signal spectrum.

**Figure 3:**
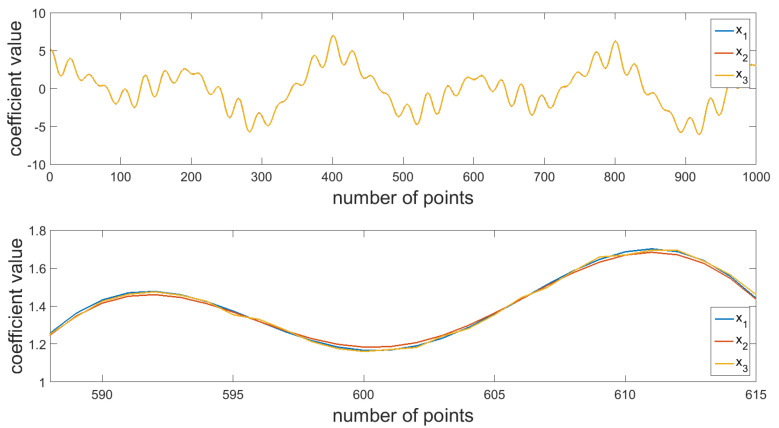
Analyzed surface profiles—simulated tests.

The tests were implemented using the concept of combining the Fourier transformation and the wavelet transformation. The paper presents a comparative analysis of various types of signals. To this end, simulation tests were performed for signals being a composition of periodic functions with various amplitude sizes and various periods (x_1_, Equation (1), Figure 3), with noise from a periodic function with a relatively small amplitude and period (x_2_, Equation (2), Figure 3), and a signal with noise from a series of coefficients with random values (x_3_, Figure 3). The functions presented in Figure 3 are almost identical, differing only in additional relatively small disruptions, which cannot be distinguished in a visual manner.

(1) $$x_{1}\left(t\right)=2\sin\left(6\pi t\right)+3\cos\left(10\pi t\right)+0.3\cos\left(30\pi t\right)+0.7\cos\left(60\pi t\right)+\cos\left(75\pi t\right)+0.07\cos\left(90\pi t\right)$$

(2) $$x_{2}\left(t\right)=x_{1}\left(t\right)+0.018\cos\left(100\pi t\right)$$

**Figure 4:**
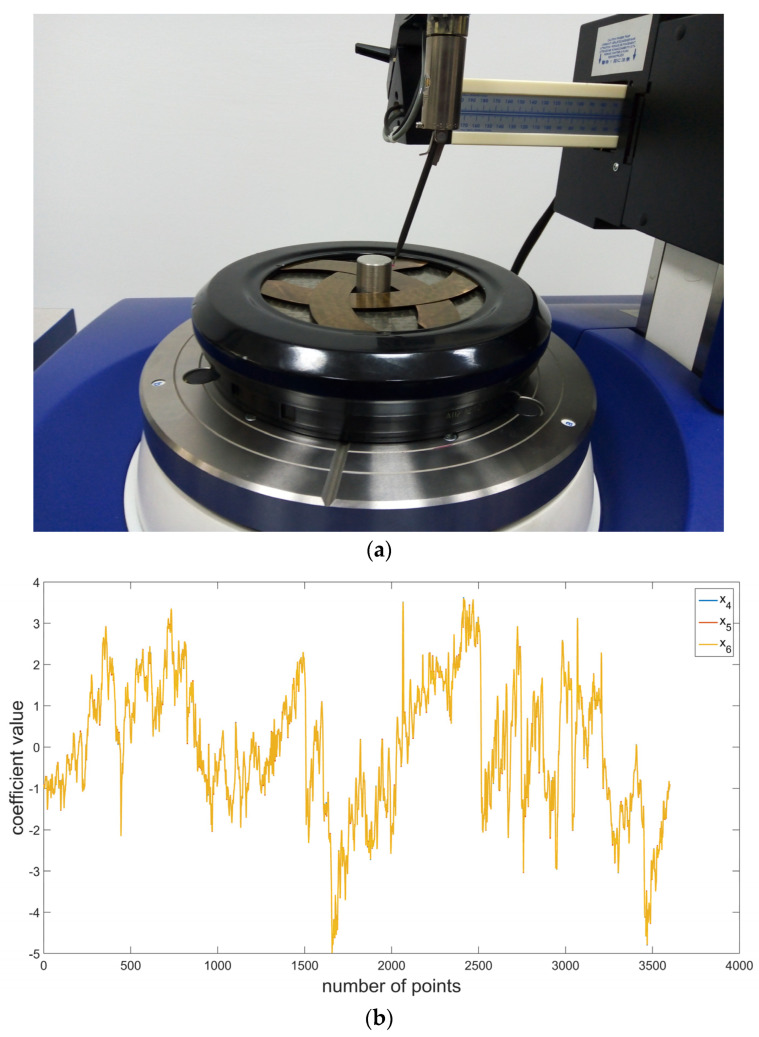
(**a**) A Talyrond 365 measurement of roundness profiles in a bearing roller and (**b**) a roundness profile with waviness components.

Experimental tests were performed to verify the results of simulation tests. Measurements of the roundness profiles of a series of rollers in rolling bearings were performed with the use of the Talyrond 365 measurement system. It is a precise measurement system; the operating principle of which is based on the method of measuring changes in the radius (radial method) with a rotary table. Talyrond 365 is commonly used in the bearing industry to analyze the quality of production of cylindrical elements [50,51]. On the other hand, the deviation of roundness and waviness of bearing rollers is of key significance, since its excess values cause the generation of additional vibrations of the rolling bearings. Moreover, they can also cause assembly problems. Figure 4a presents the measurement of a bearing roller using the example of Talyrond 365, while Figure 4b presents a sample-assessed roundness profile. 

## 3. Results

### 3.1. Simulated Tests

**Figure 5:**
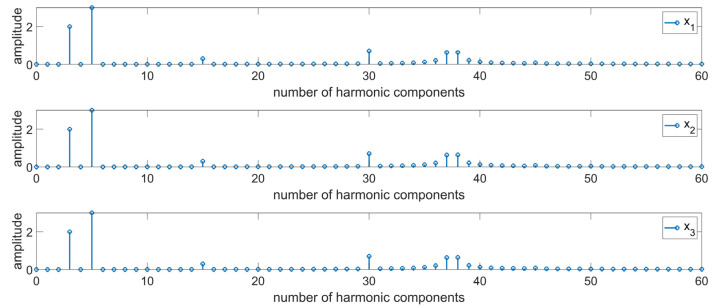
Amplitude spectral density.

A harmonic analysis was performed for the signals presented in Figure 3 (Figure 5). The resulting values of amplitudes for the individual harmonics resembling with each other, and they correspond to the modeled values of signal components. Based on either a visual assessment of the signal or the resulting values of its components, it is not possible to clearly distinguish between the individual signals. Based on the below figure, it cannot be concluded whether the signals are different, and optionally which ones of them contain additional information. Correct assessment of the signals is particularly important in terms of diagnosing a process or a product. At the initial stage of the destabilization of production processes in the individual signals received from machines and devices, there are indeed low resulting distortions, deviations from the nominal signal which should be identified as fast as possible in order to prevent deterioration of the process, which directly affects the quality of the manufactured elements. Therefore, it can be concluded that the use of the Fourier transformation only results in the lack of certain information about the signal in the produced results. The use of solely the Fourier transformation causes the loss of certain important features.

**Figure 6:**
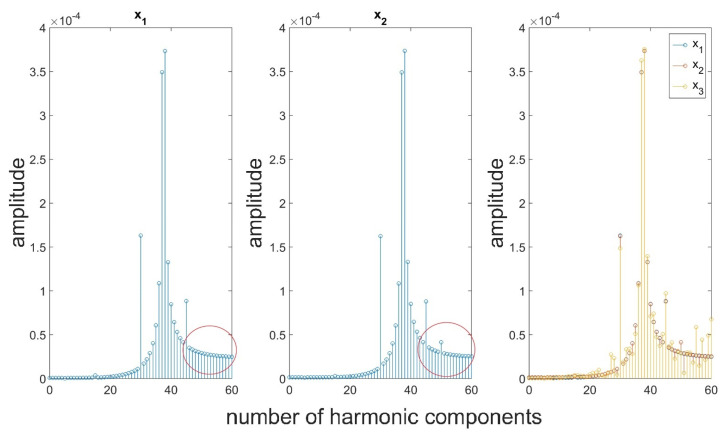
A spectrum of signals on the first level of decomposition.

**Figure 7:**
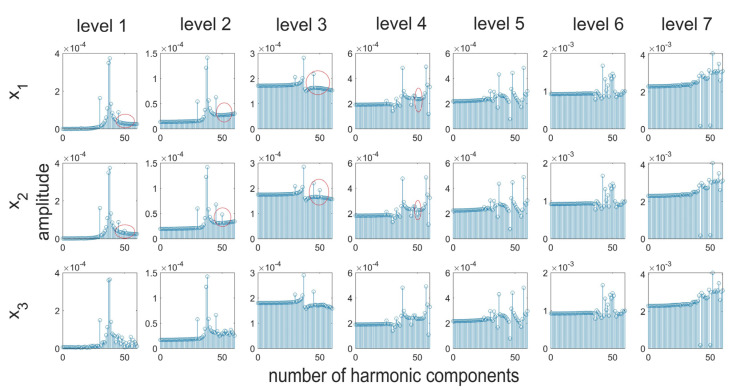
A spectrum of signals for seven levels of analysis.

In order to streamline the diagnostic process and obtain better, more precise, and comprehensive (with a broader range) information about the investigated signal, it was deemed purposeful to perform an analysis on many scales by means of the wavelet transformation. The use of the discrete wavelet transformation followed by the Fourier transform will allow for analyzing the signal in its individual frequency bands. This will allow for focusing more on high-frequency information about small amplitudes which have been lost in the spectrum presented in Figure 5. In order to perform the analysis, the db2 mother wavelet was chosen, which due to its short support width, should be sensitive to small but rapid changes in the values of the coefficients describing the signal. Figure 6 presents the results of the analysis of details generated at the first level of decomposition of signals, while Figure 7 presents the results of filtration for seven levels. Red color indicates differences in the spectrum for particular signals.

An assessment of the resulting signal spectra indicated that there are components that have been made additionally visible in the signal spectra of details created after the performance of the wavelet analysis. For each of the assessed signals, at the initial stages of the analysis, different values were obtained for certain harmonic components. When assessing the possibilities of detecting a disturbance in the form of a periodic function, it should be concluded that a disturbance corresponding to the parameters of the additional component of the signal was noticed in high-frequency signals at the four initial levels of the analysis. Therefore, on this basis, it is possible to draw conclusions about its frequency. At the following levels of decomposition, the spectra for signals x_1_ and x_2_ do not exhibit any significant differences. The additional component has been filtered out. Analogical conclusions can be drawn based on an analysis of the noisy signal (x_3_). In this case, differences between the nominal (x_1_) and the noisy signal can be noticed in a broader range of harmonics in the assessed spectra. Nonetheless, the nature of the disruption is indicated by the fact that most disruptive components were filtered out at the first stage of the analysis. Moreover, there is a considerable difference in the resulting values of spectrum coefficients at the two initial levels of the analysis.

### 3.2. Analysis of Roundness Profiles 

Analogical tests were also performed for the actual roundness profiles of rollers in a rolling bearing (x_4_). The sample surface profile presented in Figure 4b is characterized by a completely different, irregular, and aperiodic distribution of irregularities. In order to assess the possibilities of the proposed approach, it was also deemed reasonable, analogically to the case of simulation tests, to add to the analyzed profile an additional periodic function with a relatively small amplitude and period (x_5_), and a signal defined as a series of coefficients with random values (x_6_). For a comprehensive assessment, thirty roundness profiles were selected for the tests.

**Figure 8:**
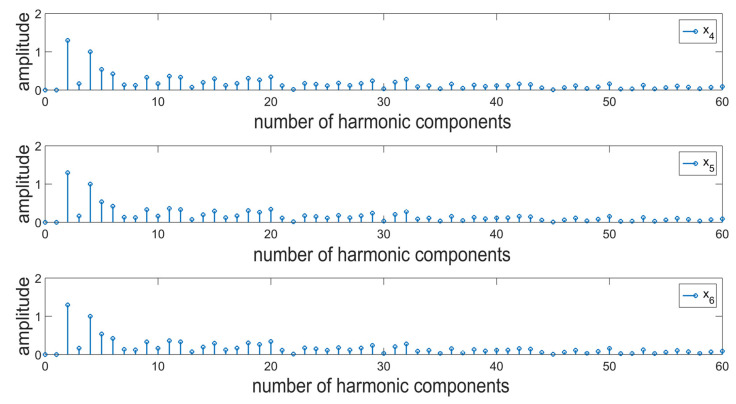
Amplitude spectral density of the roundness profiles of rollers in rolling bearings.

A harmonic analysis was performed for the sample profile presented in Figure 3 (Figure 8). The introduction of additional components in the form of a periodic function or noise caused no noticeable changes in the values of amplitudes in the resulting spectra. Based on the resulting data, it is not possible to clearly distinguish between the individual signals. The detection of changes and any deviations from the nominal assumptions is particularly important in the case of the bearing industry. It should be noted that an erroneous interpretation of the predominant harmonic in the roundness profile of a bearing roller can result in the wrong assessment of the stability of the production process. In this case, unnecessary correction procedures can be introduced, which may result in the propagation of the already existing deviations. Moreover, the excess deviation values for roundness and surface waviness of bearing rollers cause the generation of additional vibrations and noises in the rolling bearings, which is an unpreferable phenomenon [52,53]. Due to this, in the case of surface profiles, a combination of two transformations was used in order to diagnose surface topography.

**Figure 9:**
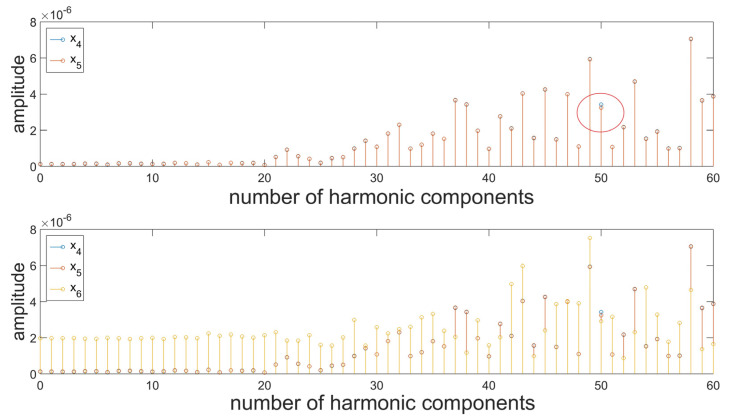
A spectrum of surface profiles on the first level of decomposition—the db2 wavelet.

**Figure 10:**
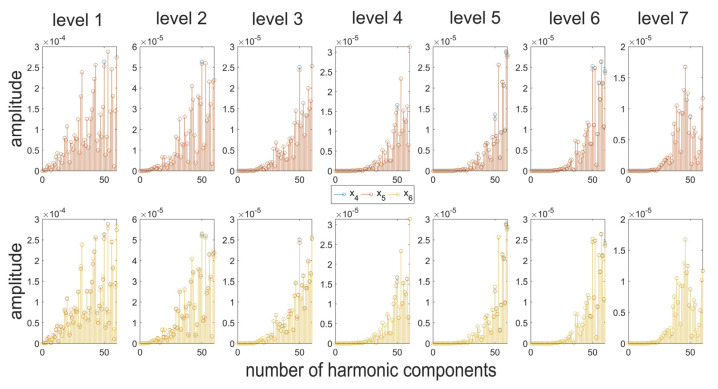
A Spectrum of surface profiles for seven levels of analysis—the db1 wavelet.

Surface profiles being a composition of periodic and aperiodic irregularities in the generated spectrum are defined by a significantly higher number of harmonic components. The assessment of irregularities in the individual bands will allow for narrowing down the scope of the analysis, which at the initial stages of decomposition results in an analysis of fast-changing information corresponding to the introduced disruptions. Figure 8 presents the results of the analysis of details generated at the first level of decomposition of signals, performed by means of the db2 wavelet. In this case, differences in the amplitude values were recorded for a single harmonic corresponding to the value of the modeled function. In the case of noise, a similar distribution of amplitude values can be observed; however, the values are proportionally higher (Figure 9). At the subsequent stages of the analysis; the resulting spectra contain no clear information about the signal. It was, therefore, deemed reasonable to use a different mother wavelet, the support width of which is even shorter, i.e., db1. The dbN family of wavelets (Daubechies), where N refers to the number of vanishing moments and wavelet order, is characterized by 2 N-1 compact support [54]. For the db1 wavelet, information in the detail signals was acquired in a clearer manner (Figure 10). Therefore, it can be concluded that for non-stationary signals, the use of wavelets with possibly short support width results in better diagnostics of the signal. In the upper part of Figure 10, there are noticeable differences for the 50th harmonic between the surface profile resulting from the measurements and the signal resulting from the addition of an additional function. There is also a noticeable level of decomposition, for which the additional component has been removed. In the case of noise, it is also possible to record the level of decomposition for which it has been filtered out. Since the wavelet analysis is a lossless transformation [55], the signal can be reconstructed, and the possible causes of the resulting defect can be analyzed to a given level.

## 4. Discussion

The continuing industrial revolution and the dynamically developing industry necessitate an increasingly accurate and comprehensive assessment of the process and the quality control of the final elements. Modern production technologies entail modern methods for analyzing measurement data. The currently widely developed multiscale methods constitute significant supplementation of the traditional algorithms used for the metrological assessment of measurement data. One of these methods is the wavelet transformation. The combined use of the wavelet and Fourier transformations allowed for extending the range of information about the assessed roundness profiles. The method can be useful in diagnostic processes for fast and comprehensive evaluation of surface topography irregularities. Feature extraction, detection of small signal changes, and additional relatively small components in signals/profiles, which in the further operation of the mechanisms can cause a real reduction in efficiency, durability, and applicability of particular elements, is a real benefit of this solution. The tests fill a research gap in terms of diagnostics and assessment of profiles, which is very important from the standpoint of the precision industry. 

Understanding the possibilities of detecting individual morphological features and determining the scale on which the irregularities occur required a comprehensive assessment and verification of the assumed procedure. The combination of both algorithms resulted in the identification and the possibility of a detailed analysis of the important aspects of geometry, which could influence the subsequent operation of the elements of machine parts. In this aspect, the traditional perception of roundness profiles seems insufficient.

The tests indicated vast potential application possibilities for multiscale methods in order to supplement the currently used traditional methods. A multiscale analysis using discrete one-dimensional wavelet transformation indicated both the prevalent components of surface irregularities, as well as additional components which had not been properly filtered out based on the Fourier transformation. An assessment of the distribution of irregularities on multiple scales allowed for better diagnostics of the roundness profiles, taking into account the scale of the individual features. The tests performed for a wide spectrum of mother wavelets and roundness profiles allowed for verifying the impact of the mother wavelet and its properties on the filtration process, positively verifying the adopted concept. Moreover, no significant diversity of the resulting values of the spectrum was obtained as a function of profile change.

The conducted tests also came with certain limitations, to be analyzed as part of research in the future. In particular, future studies should focus on a greater diversity of surface types, in particular functional surfaces, and on taking into account a broader range of types of wavelet transformations, since the individual approaches and types of the mother wavelet are characterized by different properties. This results in highlighting other characteristic morphological features on the surface [56,57,58]. In future research, it is planned to include machine parts that will contain certain surface irregularities created after a certain work cycle. The research will help to develop modern diagnostic systems allowing for fast control of the key produced industrial elements.

## 5. Conclusions

The paper assesses the possibilities of utilizing modern filtration methods for diagnosing signals. The tests were performed both for simulated irregularities distributions, as well as for the actual roundness profiles of rollers in rolling bearings. The application of an algorithm is a combination of two currently used signal analysis methods, i.e., the Fourier transformation and the wavelet transformation, which results in a better, more comprehensive analysis of data. The proposed hybrid algorithm allows for the detection of signal components that are invisible in the spectrum created in the case of performing the analysis using the Fourier transformation only. The combination of the two methods allows for providing additional information about the characteristic harmonics in the signal. There is a possibility to identify the disruptions and the occurrence of noise, as well as the parameters which describe them, which are hindered when using the Fourier transformation only. The individual values can be noted in the figures presented in the paper, where the resulting differences in spectrums are marked. Differences in amplitude values were noted for individual numbers of harmonic components, as shown, for example, in the values of the fiftieth harmonic component. Moreover, it has been noticed that the use of wavelets with a shorter support width result in better, clearer filtration at the initial stages of decomposition of additional high-frequency disrupting components. The tests performed for numerous profiles with a diverse distribution of irregularities indicated that the resulting spectra contained important additional modeled components, which were properly identified, which proves the effectiveness of the proposed approach.

## Data Availability

The data presented in this study are available upon request from the corresponding author.

## Nomenclature

x_1_	A function that is a composition of periodic functions with various amplitude sizes and various periods.
x_2_	A function that is a composition of periodic functions with various amplitude sizes and various periods, with additional noise from a periodic function with a relatively small amplitude and period.
x_3_	A function that is a composition of periodic functions with various amplitude sizes and various periods, with additional noise from a series of coefficients with random values.
x_4_	The roundness profile of rollers in a rolling bearing.
x_5_	The roundness profile of rollers in a rolling bearing, with additional noise from a periodic function with a relatively small amplitude and period.
x_6_	The roundness profile of rollers in a rolling bearing, with additional noise from a series of coefficients with random values.
N	The number of vanishing moments.
